# Reliability and Validity of the Beijing Version of the Montreal Cognitive Assessment in the Evaluation of Cognitive Function of Adult Patients with OSAHS

**DOI:** 10.1371/journal.pone.0132361

**Published:** 2015-07-24

**Authors:** Xiong Chen, Rui Zhang, Ying Xiao, Jiaqi Dong, Xun Niu, Weijia Kong

**Affiliations:** Department of Otolaryngology, Union Hospital, Tongji Medical College, Huazhong University of Science and Technology, Wuhan, China; University of Rome Tor Vergata, ITALY

## Abstract

**Background:**

The patients with obstructive sleep apnea hypopnea syndrome (OSAHS) tend to develop cognitive deficits, which usually go unrecognized, and can affect their daily life. The Beijing version of the Montreal cognitive assessment (MoCA-BJ), a Chinese version of MoCA, has been used for the assessment of cognitive functions of OSAHS patients in clinical practice. So far, its reliability and validity have not been tested. This study examined the reliability and validity of MoCA-BJ in a cohort of adult OSAHS patients.

**Methods:**

152 OSAHS patients, ranging from mild, moderate to severe, 49 primary snoring subjects and 40 normal controls were evaluated for cognitive functions by employing both MoCA-BJ and the Mini Mental State Examination (MMSE). Forty of them were re-tested by MoCA-BJ 14 days after the first test. Internal consistency, test-retest reliability, discriminate and concurrent validity of MoCA-BJ were analyzed.

**Results:**

Internal consistency reliability by Cronbach’s alpha was adequate (0.73). Intra-class correlation coefficient (ICC), an measure of test-retest reliability, was 0.87 (P<0.001). The total MoCA-BJ scores were significant higher in normal controls than in OSAHS groups (p<0.05). The performances of visuospatial ability in severe OSAHS group were significantly weaker than in normal controls and primary snoring group. The performances of executive ability in severe OSAHS patients were weaker than in normal controls. An optimal cut-off between normal controls and non-normal subjects was at 26 points (total MoCA score). Moreover, cut-off between non-severe and severe OSAHS was at 2 points on visuospatial subscale. Analysis of the correlation between MoCA total scores and MMSE total scores revealed a statistically significant, though relatively weak, correlation (r=0.41, P<0.05).

**Conclusion:**

In conclusion, our study showed that the Beijing version of the MoCA was reliable and stable. The MoCA-BJ was capable of detecting cognitive dysfunction by visuospatial and total MoCA-BJ score.

## Introduction

Obstructive sleep apnea hypopnea syndrome (OSAHS) represents a common disorder that is associated with partial or complete collapse of the upper airway during sleep [1,2] and characterized by intermittent hypoxia and repeated arousals. OSAHS may increase the risk for hypertension and cardiovascular events and cause cognitive impairment [3,4].

A great many studies examined cognitive impairments by employing both objective and subjective techniques in OSAHS. Simple MRI and functional MRI have revealed a number of specific cerebral damages in hippocampus, anterior prefrontal gyri and other regions [5,6]. Moreover, a wide array of subjective tests, including the trail making test, digit span test and the verbal fluency test, have been used for the assessment of the cognitive performance of subjects with sleep apnea [7–9]. Many people can't afford objective methods due to the cost involved. On the other hand, the subjective neuropsychological assessments involve completing a large battery questionnaires and are hard-to-use, time-consuming and intimidating to patients. In view of these problems, an easy and effective method is needed for the early detection of cognitive deficits in OSHAS patients.

The Montreal Cognitive Assessment (MoCA) developed by Nasreddine is a brief and useful screening tool with high sensitivity and specificity for detecting mild cognitive impairment [10]. It has been effectively applied for the evaluation of the cognitive impairments in many diseases such as cardiovascular disease and Parkinson’s disease [11, 12]. The MoCA was introduced into China in 2006. Translated into Chinese and tailored to local context, it was employed nation-wide for the screening of cognitive function in a large population of Chinese subjects [13,14]. So far, five Chinese versions of MoCA are available in China and the Beijing version (MoCA-BJ) has been most widely used in mainland China. Though the MoCA has been used for the cognitive assessment of OSAHS patients, its reliability and validity, to our knowledge, have not been reported [15, 16].

In this study, we used MoCA-BJ to evaluate the cognitive functions of OSAHS patients and compared them with age- and educational-background-matched non-OSAHS subjects (including normal controls and primary snoring subjects). Additionally, the concurrent validity of MoCA-BJ was evaluated against the Mini Mental State Examination (MMSE), the most commonly used cognitive screening test in the world [17]. The aim of this study was to test the reliability and validity of the MoCA-BJ and to explore the possibility of using it for the cognitive assessment of Chinese adult patients with OSAHS.

## Materials and Methods

### Ethics statement

The study was approved by the Ethics Committee of the Union Hospital of Tongji Medical College, Huazhong University of Science and Technology and written informed consent was obtained from each participant.

### Participants

A total of 201 patients and 40 age- and educational-background-matched normal controls were included in the study. The former were outpatients visiting our sleep clinics at Union Hospital of Tongji Medical College, Huazhong University of Science and Technology, Wuhan, China. Forty normal controls, who neither reported snoring nor suffered from daytime sleepiness, were selected from hospital employees and their relatives or friends.

All of them received overnight polysomnography (PSG). Before PSG, the subjects had been evaluated for cognitive function by using MoCA-BJ and MMSE. 14 days after the first evaluation, 40 of the subjects were randomly selected for re-assessment with MoCA-BJ. The Epworth sleepiness scale was employed to assess the daytime sleepiness.

Exclusion criteria for all participants: (i) history of having received treatment for sleep-related breathing disorders; (ii) presence of other sleep disorders (International Classification of Sleep Disorders (ICSD-II) diagnosis) [18], such as central sleep apnea, insomnia, restless legs syndrome, REM sleep behavior disorder; (iii) other diseases such as CNS disease, cancer, severe obstructive pulmonary disease, severe physical disabilities and mental disorders; (iv) history of having undergone major surgery within the past three months; (v) recent history of having alcoholism and being on psychotropic drugs.

In addition, before MoCA-BJ and MMSE tests, demographic data, medical history, current medications, recent mental status, lifestyle and family history were also obtained from all the subjects.

### Montreal Cognitive Assessment

The MoCA is a one-page 30-point test, which contains the following cognitive domains: visuospatial, executive, sustained attention, concentration, working memory, short-term memory recall, language and orientation. To correct for effects of education, one additional point (1 point) was awarded to MoCA total score of subject who had received only ≤12 years of education [10].

### Mini-Mental State Examination

The Chinese version of the MMSE questionnaire consists of several subscales: orientation, immediate and short-term memory recall, attention/calculation, language and visuospatial skills. Cognitive deficit on the MMSE is defined as a score less than 27 [19].

### Epworth sleepiness scale (ESS)

The ESS is a self-administered questionnaire that is widely used for subjective assessment of daytime sleepiness. It contains eight items involving eight daily-life scenarios, with each item being assessed on a 0-to-3 scale. The total scores of ESS range from 0 to 24. The cutoff point for excessive daytime sleepiness is set at >10 [20].

### Polysomnographic recordings

All patients received overnight PSG (Alice 5, Respironics, Herrsching, Germany), including electroencephalography (C3/A2, C4/A1, O1/A2, O2/A1), electrooculography, submental electromyography, bilateral anterior tibialis electromyography, electrocardiography, nasal airflow measurement, monitoring of thoracoabdominal movements, oxygen saturation, snoring and body position. Sleep stages and respiratory events were analyzed against the Sleep Medicine Criteria (American Academy, 2007) [21]. An apnea was defined as complete stoppage of airflow for at least 10 s. A hypopnea was defined as reduction in airflow by over 50% from baseline for at least 10 s with accompanying drop in arterial oxygen saturation of at least 4% and/or an electroencephalographic arousal. Finally, scoring was carried out independently by two experienced technicians who were blind to the study. The 201 non-normal subjects in our series were divided into four groups: primary snoring group was made up of subjects with AHI<5 /hr, mild OSAHS group contained subjects with 5≤AHI<15 /hr; moderate OSAHS group was comprised of those with AHI ranging between 15 and 30/hr; and severe OSAHS group was consisted of subjects with AHI≥30/hr. In normal controls, the AHI was <5 /hr[21].

### Statistical analyses

All analyses were performed by means of SPSS software package (version 19.0) (SPSS Inc, Chicago, IL, USA) and Medcalc version 15.2.2 (Medcalc Software, Ostend Belgium). The distribution of the data was analyzed by employing the Kolomogorov-Smirnov test. Data were presented as mean±standard deviations for continuous variables with normal distribution. Medians and interquartile ranges were used for continuous variables without normal distribution and categorical variables.

To test the differences in the demographic, clinical, primary polysomnographic parameters and cognition scores among five groups, one-way analysis of variance (ANOVA) and Kruskal-Wallis were performed. Internal consistency reliability was tested in terms of Cronbach's alpha coefficient and subscale-total correlation coefficient. Test-retest reliability was evaluated in terms of intraclass correlation coefficients (ICC). Spearman correlation coefficient was used to test concurrent validity between the MoCA-BJ total scores and MMSE total scores. The Spearman correlation analysis and multiple regression analysis were performed to assess correlations between the parameters (age, education, BMI, AHI, the minimum oxygen saturation (L-SaO_2_), mean oxygen saturation (A-SaO_2_) and the time spent at SaO_2_<90% (CT90)) and total scores of MoCA-BJ and its subscales scores.

To further determine the discriminate validity of the MoCA-BJ in OSAHS, receiver operating characteristic (ROC) curves of MoCA-BJ and all the cognitive domains were generated among the five groups. The area under the curve (AUC) was calculated for each ROC curve. The cutoff score, optimal sensitivity and specificity were determined on the basis of Youden index.

Statistical significance for all analysis was set at a P value < 0.05.

## Results

### Demographic data and clinical findings

The age of the participants ranged from 18 to 55 years, with their median time of education being 14 and 16 years (range: 6 to 16 years). The details of sex distribution, BMI, ESS scores, AHI, L-SaO_2_, A-SaO_2_ and CT90 were listed in Table 1. The rates of hypertension ranged from 0% to 11.76% and 17.65% among groups. Smoking rates ranged from 21.3% to 24.5% and 43.14% among groups.

**Table 1:** Demographics and Clinical data for studied participants.

	Normal control	Primary snoring	Mild OSAHS	Moderate OSAHS	Severe OSAHS	P
Number	40	49	51	50	51	
Age(years)^a^	34.53±9.95	32.53±10.20	33.33±7.97	35.40±10.43	32.80±9.94	>0.05
Male, number(%)	62%	51%	74.5%	76%	76.5%	
BMI(Kg/m^2^)^a^	21.92±2.02 ^c^	22.49±3.25 ^c^	24.76±3.15 ^d^	25.45±3.29 ^d^	26.38±4.52 ^d^	<0.05
Education(years) ^b^	14(4)	16(4)	16(4)	16(4)	16(4)	>0.05
Smoking number(%)^a^	21.30%	24.50%	39.22%	38.00%	43.14%	
Hypertension(mmHg)	0%	4.1%	11.76%	6%	17.65%	
ESS ^a^	2.08±2.19	8.84±5.15 ^e^	9.67±5.31 ^e^	9.42±4.83 ^e^	11.71±5.93	<0.05
AHI (events/h)^a^	2.80±1.28 ^f^	2.65±1.40 ^f^	10.12±2.89	21.70±4.43	55.62±18.27	<0.05
Minimum SaO_2_ (%)^a^	91.47±1.74 ^g^	89.84±4.89 ^g^	85.34±12.56	80.02±12.37	72.57±10.66	<0.05
Average SaO_2_ (%)^a^	95.55±1.35 ^h^	96.05±1.78 ^h^ ^,^ ^i^	96.18±1.38 ^h^ ^,^ ^i^	94.78±1.72 ^h^	92.28±5.50	<0.05
Time at SaO_2_<90% (%)^a^	0.00±0.00 ^j^	1.00±5.60 ^j^	0.00±0.60 ^j^	5.00±10.10	19.00±21.00	<0.05

^a^ Variables are normal distributed. Results are presented as mean±standard deviation. p values were calculated with ANOVA test.

^b^ Variables are non-normal distributed. Results are presented as median and interquartile range. p values were calculated using Kruskal-Wallis test.

^c^ p>0.05, primary snoring vs normal control.

^d^ p>0.05, moderate OSHS vs mild, severe OSAHS.

^e^ p>0.05, primary snoring vs mild vs moderate OSAHS.

^f,g^ p>0.05, normal control vs primary snoring.

^h^ p>0.05, normal control vs primary snoring, mild and moderate OSAHS.

^i^ p>0.05, primary snoring vs mild OSAHS.

^j^ p>0.05, normal control vs primary snoring vs mild OSAHS

ESS, Epworth Sleepiness Scale AHI, Apnea–hypopnea index, SaO_2_, oxygen saturation, OSAHS, obstructive sleep apnea hypopnea syndrome

### Internal consistency and test-retest reliability

The Cronbach’s alpha of the MoCA-BJ was 0.73(p<0.05). The correlations between visuospatial, executive, attention, language, short-term memory, and orientation and MoCA-BJ were 0.65, 0.68, 0.42, 0.56, 0.67 and 0.16, respectively. Forty subjects were retested 14 days after the first interview, and the test-retest reliability was 0.87 (95% confidence interval [CI] = 0.77–0.93, P < 0.001).

### Validity, sensitivity and specificity of the MoCA-BJ and related factors

The total MoCA-BJ scores were significant higher in normal controls than in OSAHS groups (p<0.05). The total MoCA-BJ scores were comparable between normal controls and primary snoring group. The performances of visuospatial ability in severe OSAHS group were significantly weaker than in normal controls and primary snoring group. The visuospatial score in severe OSAHS subjects was also lower than in mild OSAHS group, but the difference was not significant (p = 0.051). No significant differences were revealed in visuospatial ability among normal controls, the primary snoring, mild and moderate OSAHS groups. The performances of executive ability in severe OSAHS patients were weaker than in normal controls. No significant differences were observed in attention, short-term memory recall, language, and orientation domains among the five groups. The results are detailed in Table 2.

**Table 2:** The results of MoCA-BJ cognitive domains and responding cognitive tasks.

	Normal control	Primary snoring	Mild OSAHS	Moderate OSAHS	Severe OSAHS
Visuospatial ^a^	4(1) ^c^	4(1) ^c^	4(1) ^d^	3(1)	3(2) ^c^ ^,^ ^d^
Cube copy ^a^	1(0) ^e^	1(0) ^e^	1(1)	1(1)	0(1) ^e^
Clock drawing ^a^	3(0) ^f^	3(0.5) ^f^	3(1) ^f^	3(1) ^f^	2(1) ^f^
Executive ^a^	4(1) ^g^	4(2)	3(2)	3(2)	3(2) ^g^
Trail making B^a^	1(0)	1(0)	1(1)	1(0)	1(0)
Verbal fluency ^a^	1(0)	1(0)	1(0)	1(0)	1(0)
Abstract ^a^	2(1) ^g^	2(1)	2(1)	1(1)	1(2) ^g^
Attention ^a^	6(0.75)	6(0)	6(1)	6(0.25)	6(0)
Figure tapping ^a^	1(0)	1(0)	1(0)	1(0)	1(0)
Digit span forward and backward ^a^	2(0)	2(0)	2(0)	2(0)	2(0)
Calculation ^a^	3(0)	3(0)	3(0)	3(0)	3(0)
Language ^a^	4(1.75)	4(1)	4(2)	4(1.25)	4(1)
Naming ^a^	3(0)	3(1)	3(0)	3(1)	3(1)
Repeat ^a^	1(1)	2(1)	1(1)	1(2)	2(1)
Short term memory ^a^	4(2)	4(1.5)	4(1)	3.5(1)	4(2)
Oritation ^a^	6(0)	6(0)	6(0)	6(0)	6(0)
MoCA total score ^b^	27.25±2.41 ^h^	26.24±2.49	26.08±2.58 ^h^	25.42±2.60 ^h^	25.37±3.34 ^h^

^a^ Variables were non-normal distributed. Results are presented as median and interquartile range. p values were calculated using Kruskal-Wallis test.

^b^ Variables were normal distributed. Results are presented as mean±standard deviation. p values were calculated with ANOVA test.

^c^ p<0.05, severe OSAHS vs normal control, primary snoring

^d^ p = 0.051, mild OSAHS vs severe OSAHS

^e^ p<0.05, severe OSAHS vs normal control, primary snoring

^f^ p<0.05, severe OSAHS vs normal control, primary snoring, mild, moderate

^g^ p<0.05, normal control vs severe OSAHS

^h^ P<0.05, normal control vs mild,moderate,severe OSAHS.

**Fig 1:**
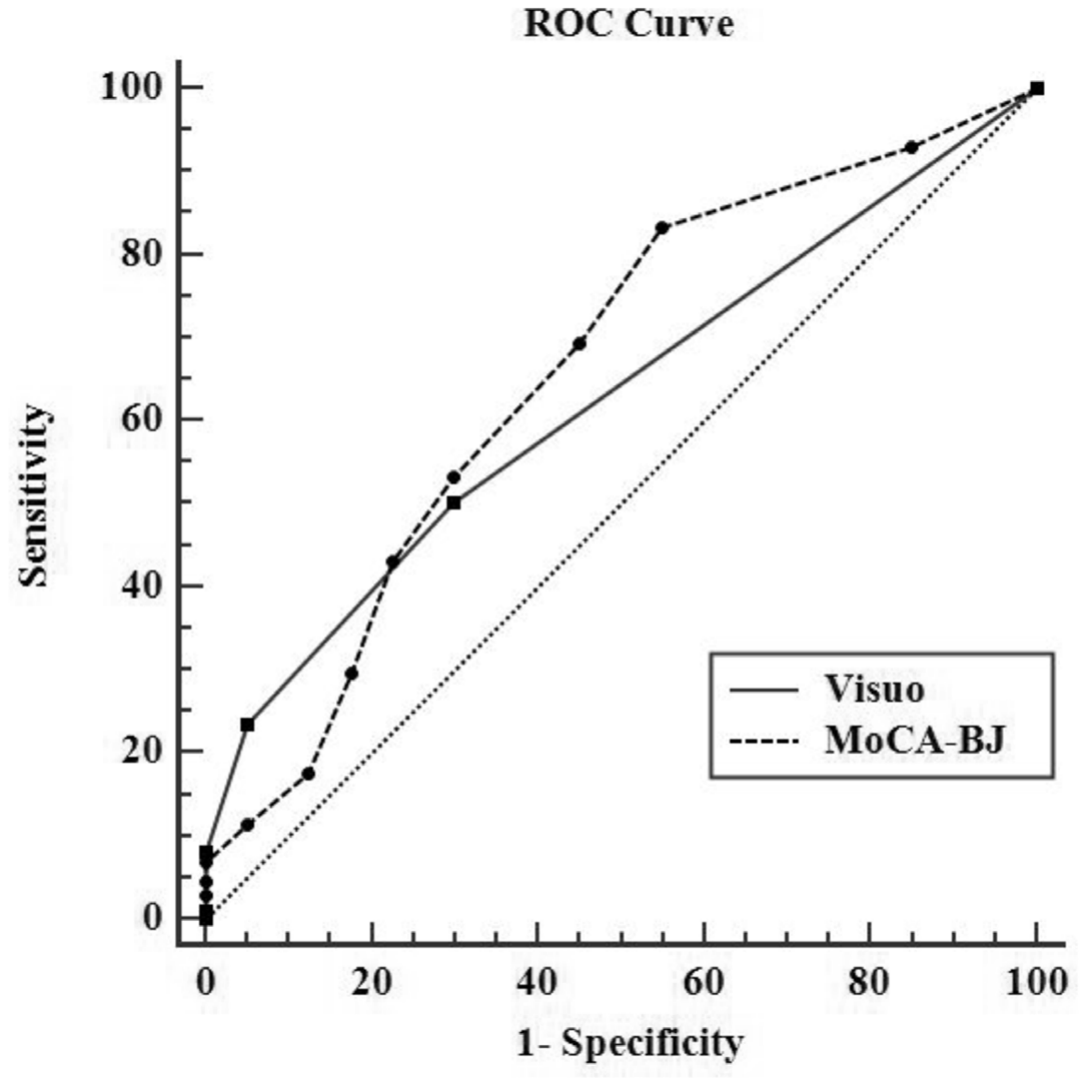
The Receiver characteristic (ROC) curves of global MoCA-BJ and visuospatial ability between normal controls and non-normal subjects. The area under the curve of MoCA-BJ was 0.66 (95% CI 0.59–0.72), an sensitivity of 54.23% and specificity 70.0%. The AUC of visuospatial subscale was 0.63 (95% CI 0.56–0.69), the sensitivity was 50.75% and specificity was 70.0%. MoCA-BJ, Beijing version of the Montreal Cognitive Assessment. Visuo: Visuospitial.

**Fig 2:**
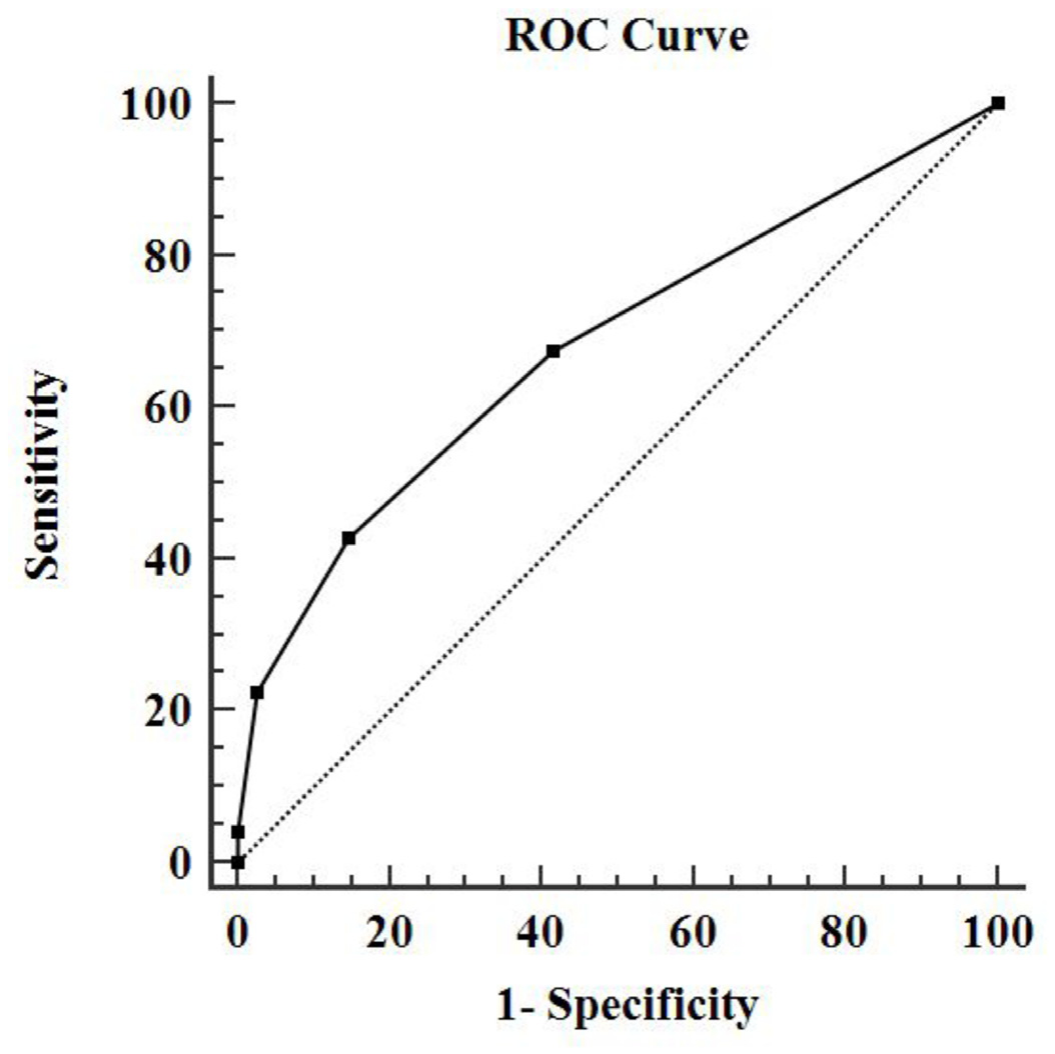
The Receiver characteristic (ROC) curve of visuospatial between non-severe subjects and severe OSAHS. The area under the curve of visuospatial ability was 0.68 (95%CI 0.62–0.74), the sensitivity was 41.18% and specificity was 85.26%.

The ROC analysis identified an optimal cut-off between normal controls and non-normal subjects at 26 points (total MoCA score), with a sensitivity of 54.23%, specificity of 70.0% and AUC of 0.66 (95% CI 0.59–0.72). The optimal cut-off for the visuospatial subscale was at 3 points, with an sensitivity of 50.75%, specificity of 70.0% and AUC of 0.63 (95% CI 0.56–0.69). Moreover, optimal cut-off between severe OSAHS and non-severe OSAHS was found to be at 2 on visuospatial subscale, with the sensitivity was 41.18% and specificity was 85.26%, AUC was 0.68 (95%CI 0.62–0.74). Details are given in Table 3, Figs 1 and 2.

**Table 3:** Discrimination validity of the MoCA-BJ (with 95% confidence interval, CI) among groups.

	AUC(95%CI)	Cut-off value	Sensitivity (95%CI),%	Specificity (95%CI),%
Normal versus Non-normal				
MoCA-BJ	0.66(0.59–0.72)	≤26	54.23(47.1–61.3)	70.0(53.5–83.4)
Visuospatial	0.63(0.56–0.69)	≤3	50.75(43.6–57.9)	70.0(53.5–83.4)
Severe versus Non-severe group				
Visuospatial	0.68(0.62–0.74)	≤2	41.18(27.6–55.8)	85.26(79.4–90.0)

AUC, area under the curve MoCA-BJ, Beijing version of the Montreal cognitive assessment

Correlation analysis between MoCA total scores and MMSE total scores revealed a statistically significant, though relatively weak correlation (r = 0.41, P<0.05). There were no significant differences in MMSE total scores and sub-domain scores among the five groups. The results are listed in Table 4.

**Table 4:** Comparisons of MMSE among groups.

	Normal control	Primary snoring	Mild OSAHS	Moderate OSAHS	Severe OSAHS	p
Time orientation ^a^	5(0)	5(0)	5(0)	5(0)	5(0)	0.86
Place orientation ^a^	5(0)	5(0)	5(0)	5(0)	5(0)	0.44
Immediate memory ^a^	3(0)	3(0)	3(0)	3(0)	3(0)	0.48
Short-term memory recall ^a^	3(0.75)	3(1)	3(1)	3(1)	3(1)	0.50
Attention ^a^	5(1)	5(1)	5(1)	5(1)	5(1)	0.30
Language ^a^	3(0)	3(0)	3(0)	3(0)	3(0)	0.11
Viuospatial ^a^	6(0)	6(0)	6(0)	6(0)	6(0)	0.48
Summary ^a^	29(1)	30(1.5)	29(2)	29(2)	29(2)	0.19

^a^ Variables are non-normal distributed. Results are presented as median and interquartile range. P values were analyzed by Kruskal-Walli tests.

MMSE:Mini Mental State Examination

The associations between four polysomnographic measures of OSAHS severity (AHI, L-SaO_2_, A-SaO_2_ and CT90) and MoCA-BJ total scores and subscale scores were analyzed. It was found that the MoCA total score was significantly correlated with L-SaO2 (r = 0.16,β = 0.18), and the visuospatial skill was significantly correlated with AHI (r = 0.29,β = 0.21). There existed no significant correlations between other subscales scores and AHI, L-SaO_2_, A-SaO_2_ and CT90. As to anthropometric variables, the age educational level were found to be significantly correlated with many subscales of MoCA-BJ, the details were listed in Table 5. However, the gender distribution and BMI were not significantly correlated with the total scores and subscales scores of MoCA-BJ.

**Table 5:** The correlations between age, education and MoCA-BJ cognitive domains and the correlation between cognitive domains and MoCA-BJ total score.

	Age	Education	MoCA-BJ	P
Visuospatial	0.13	0.38	0.65	< 0.05
Executive	0.25	0.37	0.68	< 0.05
Attention	-	0.19	0.42	< 0.05
Short-term memory recall	0.14	0.17	0.67	< 0.05
Language	0.24	0.19	0.56	< 0.05
Orientation	-	-	0.16	< 0.05
MoCA-BJ	0.28	0.27	-	< 0.05

MoCA-BJ, Beijing version of the Montreal Cognitive Assessment

## Discussion

In this study, we found that the Beijing version of the Montreal Cognitive Assessment possessed adequate internal consistency and good test-retest reliability. OSAHS patients performed poorly on global MoCA-BJ than normal controls. The severe OSAHS also had poor performance on tests of visuospatial and executive ability. According to the results from ROC curve, the MoCA-BJ demonstrated adequate ability to discriminate normal controls from non-normal subjects and severe OSAHS from non-severe OSAHS.

Internal consistency of the MoCA-BJ (Cronbach’s alpha = 0.73) satisfied the recommended value for internal consistency and was similar to those of previous studies conducted in some non-English speaking populations [22, 23]. Among the subscales in the MoCA-BJ, almost all correlations were above the minimum recommended criterion for adequate fit, except that of the orientation (r = 0.16<0.3), suggesting that the subscale orientation can be excluded when MoCA-BJ is employed for the assessment of cognitive function in adult OSAHS. The high test-retest reliability demonstrated that the MoCA-BJ is stable over time.

On global MoCA-BJ test, OSAHS patients preformed more poorly than normal controls. The total MoCA-BJ scores did not differ among primary snoring, mild, moderate and severe OSAHS groups. Our findings were consistent with the result of one previous study in which comparison was made between only two groups (OSAHS and healthy controls)[16]. Moreover, the total MoCA-BJ scores were found to be associated with L-SaO_2_. Our findings were consistent with the findings of a recent study, which revealed that total MoCA-BJ scores was correlated with L-SaO_2_ in OSAHS [15].

In this study, cube copy and clock drawing tests showed that visuospatial ability was reduced in severe OSAHS group. Our findings were similar to the results reported by Greenberg and Bédard who compared normal controls and subjects with sleep apnea in terms of visuospatial/construction ability by using Copy test. They found that sleep apnea patients performed less well than controls [1, 24]. Pietrini *et al* also found, by employing Drawing test, the visuospatial/construction ability was reduced in sleep apnea [25]. Furthermore, we found that the AHI was one contributor to the visuospatial ability impairment. As to whether disease severity and visuospatial capacity are related, different studies reached different conclusions. Some studies reported the visuospatial/construction ability was associated with sleep apnea severity, while some studies did not found associations [24,26,27]. The discrepancies might be ascribed to differences in methods used, subjects included and severity of the diseases.

In this study, we found that the severe OSAHS patients had significantly poorer performance on abstract test, which measures executive functions, than normal controls and no significant differences were found in trail making B and verbal fluency between severe OSAHS patients and normal controls. This findings were similar to some previous studies [28,29]. However, some studies found the people with sleep apnea had executive dysfunction on tests of trail making B and verbal fluency when compared with controls [1,30]. Since psychometric methods and study designs varied, even the same tests were used, differences among groups might or might not be revealed.

The global MoCA-BJ demonstrated adequate ability to discriminate normal controls from non-normal subjects. The point of 26 (global MoCA-BJ) might be a cutoff to differentiate normal controls from non-normal subjects. Moreover, visuospatial scale was demonstrated to be adequately able to distinguish the non-severe OSAHS from severe OSAHS. The point of 2 on visuospatial subscale might be the cutoff to discriminate non-severe OSAHS from severe OSAHS.

Many studies reported that patients with sleep apnea tended to suffer from impaired attention/vigilance. Nonetheless, in this study, no differences were found in attention among the five groups. Attention involves a number of sub-domains and a great many tests for attention are available. In previous studies, the four-choice reaction time (FCRRT) and the psycho-motor vigilance (PVT) tests showed that the sustained attention was impaired in patients with sleep apnea [31–33] and the Stroop test exhibited that subjects with sleep apnea had poorer selective attention ability [34,35]. In our study, the tasks of finger tapping, digit span and calculation in MoCA-BJ were not sensitive enough for the detection of the attention impairment. Despite low sensitivity of above tasks, the cube copy, drawing, abstract, and global MoCA-BJ scores, when used in combination, can effectively measure cognitive ability.

The short-term verbal memory recall process was tested in MoCA-BJ, no differences were found among groups. Memory processes are involved and previous studies, using WMS: figural memory and Rey-Osterrieth figure tasks, found that the long-term memory recall was impaired in sleep apnea subjects [25,32]. The Benton Visual Retention Test (BVRT) and WMS: logical memory tests showed that the immediate recall memory ability did not differ between patients and controls [1,4]. Beebe and colleges, in their meta-review study, reported that the impact of OSA on short-term verbal memory was marginal and not statistically significant [36]. It remains elusive which aspects of memory are most vulnerable to or affected by OSA. In addition, our study did not exhibit any differences in language ability among the five groups. Meta-review by Bucks and Beebe showed that language ability was not affected by OSA [36, 37].

Our study showed that the correlation between MoCA-BJ total scores and MMSE scores was weak and this might be attributed to the fact that the two instruments don’t cover exactly the same domains of cognitive function. The MMSE couldn’t differentiate adult normal subjects from primary snoring and OSAHS subjects.

Among the demographic variables, age and educational level were found to be correlated with many MoCA-BJ subdomains. Our findings were consistent with the previous studies which suggested that the MoCA scores were significantly associated with age and educational level [14, 38].

This study had some limitations. First, age of our subjects were ranged from 18–55 and the results may not be extrapolated to older or younger populations. Second, this study was not a population-based and more normal subjects from communities should be included to better meet the statistical requirements.

In conclusion, our study showed that the Beijing version of the MoCA was reliable and stable in adult OSAHS patients. The MoCA-BJ was capable of detecting cognitive dysfunction by visuospatial and total MoCA-BJ score, though it was not sensitive to attention and memory deficits. In future, research efforts should be directed at examining the relationship between MoCA-BJ and other sensitive measures and other objective tests.

## Supporting Information

S1 File The minimal data of this study. (XLS)
